# Fast, Multiphase Volume Adaptation to Hyperosmotic Shock by *Escherichia coli*

**DOI:** 10.1371/journal.pone.0035205

**Published:** 2012-04-13

**Authors:** Teuta Pilizota, Joshua W. Shaevitz

**Affiliations:** 1 Lewis-Sigler Institute for Integrative Genomics, Princeton University, Princeton, New Jersey, United States of America; 2 Department of Physics, Princeton University, Princeton, New Jersey, United States of America; Arizona State University, United States of America

## Abstract

All living cells employ an array of different mechanisms to help them survive changes in extra cellular osmotic pressure. The difference in the concentration of chemicals in a bacterium's cytoplasm and the external environment generates an osmotic pressure that inflates the cell. It is thought that the bacterium *Escherichia coli* use a number of interconnected systems to adapt to changes in external pressure, allowing them to maintain turgor and live in surroundings that range more than two-hundred-fold in external osmolality. Here, we use fluorescence imaging to make the first measurements of cell volume changes over time during hyperosmotic shock and subsequent adaptation on a single cell level *in vivo* with a time resolution on the order of seconds. We directly observe two previously unseen phases of the cytoplasmic water efflux upon hyperosmotic shock. Furthermore, we monitor cell volume changes during the post-shock recovery and observe a two-phase response that depends on the shock magnitude. The initial phase of recovery is fast, on the order of 15–20 min and shows little cell-to-cell variation. For large sucrose shocks, a secondary phase that lasts several hours adds to the recovery. We find that cells are able to recover fully from shocks as high as 1 Osmol/kg using existing systems, but that for larger shocks, protein synthesis is required for full recovery.

## Introduction

At any given moment, bacteria are exposed to various external stresses including changes in temperature, the concentrations of extracellular salts and metals, and nutrient deprivation. To survive these challenges, cells rely on the ability to both sense and respond to unfavorable perturbations. Many bacteria are able to survive in a large variety of chemical environments that can vary substantially in osmolality. *Escherichia coli*, for example, can grow in conditions ranging from 5 mOsmol/kg to ∼3 Osmol/kg [1], [2]. Even more impressive, these cells can quickly survive large changes in osmolality, often recovering to full exponential growth in a matter of hours. It remains unclear how cells are able to cope with such large chemo-mechanical perturbations. Here, by looking at the volume adaptation to hyperosmotic shocks on a single cell level, we show that it proceeds in distinct phases that depend on the shock magnitude.

The difference between the intracellular and extracellular chemical compositions generates a turgor pressure in all bacteria. A gram-negative cell's fluid cytoplasm is separated from the external environment by the inner membrane, the periplasmic space and the outer membrane. Ordinarily, the total solute concentration within the cytoplasm is higher than that of the environment, resulting in a positive osmotic pressure on the cell wall [3]. This turgor pressure is thought to help maintain cell shape and growth. When the external chemical environment surrounding a cell changes, *E. coli* cells respond by adjusting their internal solute concentration using several different regulatory pathways.

*E. coli* is able to respond to both increases and decreases in external concentrations. A downward shift in external osmolality (termed hypoosmotic shock) causes water influx into the cell's cytoplasm. As a result, the turgor pressure increases and the cell expands in a nonlinear fashion [4]. An increase in membrane tension is then thought to activate the nonspecific export of solutes through mechano-sensitive channels such as MscS and MscL [5]. In contrast, an increase in external osmolality (termed hyperosmotic shock) causes water efflux from the cell interior, a reduction in turgor pressure and a reduction in the cell volume. Under these circumstances, cells respond by actively accumulating specific solutes, termed osmoprotectants, which include ions such as potassium and organic osmolytes such as proline and glycine betaine. In addition, the activation of specific biosynthetic pathways is thought to further increase the cytoplasmic osmolality.

The permeability of *E.coli's* inner and outer membrane to the passage of different solutes can be very complex [6]. For example, the outer membrane is thought to be permeable to sucrose while the inner membrane is not [6]. Thus, sucrose is able to enter the *E.coli* periplasm but not the cytoplasm. Larger sugars, such as dextrans with molecular weight above 600, have been shown not to cross either of the two membranes [6]. On the other hand, sodium chloride permeability has not been well understood due to its complexity [7], [8].

Previous work has studied various aspects of osmoregulation in *E. coli*. Several groups have probed survival, growth rate and lag phase duration after an osmotic shock [9]–[14]. It has also been possible to monitor the uptake of cytoplasmic potassium and other osmolytes as well as the water-accessible cell volume in populations of cells during adaptation [11]–[16].

Pioneering genetic studies in *E. coli* have led to the identification of many key components of the osmoregulatory network. It is now known that the Trk, Kup and Kdp transporters mediate the uptake of K^+^, while the ProP, ProU, BetT and BetU transporters mediate the uptake of organic osmolytes [17]–[19]. In addition, *in vitro* experiments using many of these proteins have probed their activity by inserting them into lipid vesicles [20]–[23].

These and other studies have largely established the molecular players involved in osmoregulation. To understand how a cell responds mechanistically to osmotic shock at the cellular level, higher temporal and spatial resolution measurements need to be made. Whole cell measurements to date have lacked a real-time probe of cell morphology, thus making the study of the short-time scale passive and active responses to osmotic shock difficult. As a result, many fundamental questions about osmoregulation such as how a shock is sensed, when recovery begins and in what temporal order the different mechanisms come into play remain concealed.

**Figure 1:**
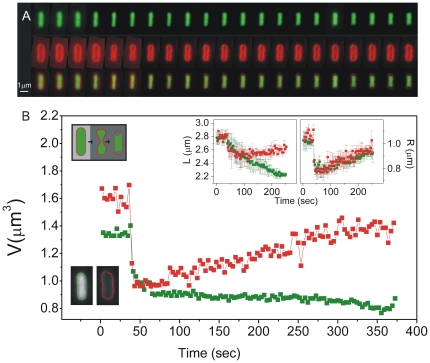
Passive response to hyperosmotic shock involves two phases of cell shrinking. (A) A sequence of fluorescent images of *E.coli* cells prior and during the hyperosmotic shock. Top: cytoplasmic volume marked with the green protein EGFP; Middle: total cell volume marked by the red outer membrane dye FM4-64; Bottom: overlay if EGFP and FM4-64. A cell is transferred from isoosmotic buffer (10 mM Tris-HCl buffer supplemented with 150 mM sucrose) to isoosmotic buffer and 620 mM sucrose. Images were acquired at a frame every 1.6 seconds alternating between the cytoplasmic volume and total cell volume. Image brightness was adjusted due to photo bleaching and the difference in fluorescence intensity between the FM4-64 and EGFP. Videos are given in Video S1 and S2. (B) Volume recovery versus time obtained using cell area analysis. Inset: cell length and cell radius versus time. Total cell volume, length and radius are given in red and cytoplasmic cell volume, length and radius in green. An example contour of the cytoplasmic cell volume and total cell volume obtained after cell area analysis for the initial frame is shown on the bottom left. The inset shows a cartoon illustration of the post sucrose shock events described in the text.

To address some of these questions, we sought to quantify cytoplasmic and periplasmic morphologies in living *E. coli* cells with high spatial and temporal resolution during osmotic shock and recovery (Figure 1). Briefly, we calculated the volume in these different cellular compartments by imaging a cytoplasmicly-expressed fluorescent protein and a fluorescent outer-membrane dye either simultaneously or individually.

This approach allows for real-time observation of both the passive water efflux response to hyperosmotic shock and the subsequent active recovery. We determined that upon hyperosmotic sucrose shock, cells shrink with two characteristic rates. The initial rate is on the order of seconds and is responsible for most of the volume reduction, however it does not depend on the *E.coli* aquaporin AqpZ.

We further measured cell volume recovery for different hyperosmotic sucrose shocks. The recovery proceeds immediately after shock and shows two distinct phases. The faster phase lasts about 20 minutes, after which the rate of recovery becomes noticeably slower. The slow phase lasts for several hours and is followed by a long pause on the order of hours during which no change in cell volume is observed. For very large hyperosmotic shocks, cells no longer utilize the fast phase as a significant recovery mechanism, but instead depend primarily on protein-synthesis-dependent recovery pathways.

## Results

### Water efflux from the periplasm and cytoplasm are separate events

To investigate the passive response of *E.coli* cells to hyperosmotic shock we simultaneously monitored a cytoplasmicly-expressed fluorescent protein and a fluorescent outer-membrane dye with a sampling rate of 0.6 Hz. The chosen field of view was maintained at a fixed *xyz*-position over time using a laser-based stabilization system (*Materials and Methods*). We isolate passive efflux from active solute transport and recovery by first transferring the cells from LB into an isotonic buffer lacking osmoprotectants prior to osmotic shock. Figure 1A shows fluorescent images of a cell prior to and after hyperosmotic sucrose shock. Previous studies predominantly use sodium chloride and occasionally sucrose as shocking media [11], [15], [24], [25]. We chose sucrose over sodium chloride because more about *E.coli's* membrane permeability to sucrose is known [6].

The cytoplasmic and periplasmic components of a cell respond differently to hyperosmotic sucrose shock in two distinct stages. Figure 1B shows the cell length, radius, total cellular and cytoplasmic volumes derived from the fluorescent images as described in the *Materials and Methods*. Based on our measurements, the periplasm occupies approximately 16% of the total cell volume and the separation between the inner and outer membranes is (45±10) nm. Electron microscopy images of the bacterial flagellar motor show that the separation between the outer membrane spanning L-ring component of the motor and the inner membrane spanning MS-ring is approximately 40 nm, in agreement with our measurements [26].

In the first stage of shrinking, cell volume decreases in a few seconds and the cells adopt an hour-glass shape (Figure 1A, middle). During this phase, both the cell radius and length decrease (Figure 1B, insets). In the second stage, which lasts for several tens of seconds, the radius of the outer membrane increases with no significant length change (Figure 1B, inset). In contrast, while the cytoplasmic radius also increases, the cytoplasmic length decreases such that the cytoplasmic volume decreases only slightly, remaining roughly constant (Figure 1A, bottom right and Figure 1B, inset). The final shape of the cell is that of a typical plasmolysed cell observed in electron microscopy in which the inner membrane separates from the cell wall primarily at the cell poles (Figure 1A, right) [24], [27]. The two-stage response of cells to sucrose shock is likely due to sucrose's ability to penetrate the periplasm but not the cytoplasm [6]. We propose that the second stage, in which the periplasmic volume increases, is due to sucrose entering the periplasmic space and allowing it to rehydrate.

### Water efflux upon hyperosmotic sucrose shock proceeds at two rates

**Figure 2:**
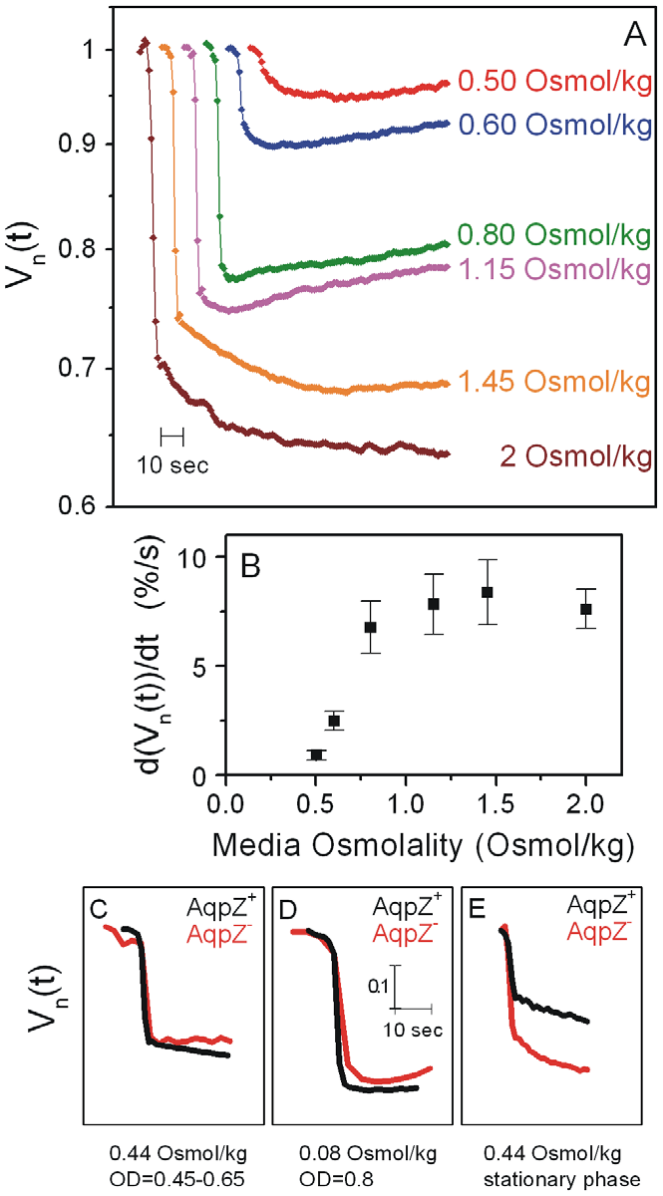
Cell shrinking is fast and doesn't require AqpZ. (A) Averaged volume traces shown for different shock magnitudes (V_n_(t)). For each cell in a given data set, the recovery trace was normalized with initial volume and aligned at the time of hyperosmotic shock. The average trace was computed from normalized and aligned data sets as described in the Materials and Methods. For display, the averaged trace for each shock is shifted 10 seconds in time compared to the last one. (B) Rate of initial fast volume reduction *dV_n_(t)/dt* for different shock magnitudes. Error bars are standard error of the mean. (C)–(E) Averaged volume traces (V_n_(t)) aquired at 1 Hz resolution for AqpZ+ strain (black) and AqpZ− strain (red). (C) Cells were grown in LB (0.44 Osmol/kg) to OD of 0.45–0.65 and transferred to LB with 620 mM sucrose. Approximately 100 cells were used for the AqpZ+ strain and 14 cells for the AqpZ− strain. (D) Cells were grown in low osmolarity LB (prepared with no NaCl to a final osmolarity of 0.08 Osmol/kg) to OD = 0.8 and transferred into low osmolarity LB and 620 mM sucrose. Averaged traces were obtained from 23 cells for ApqZ+ and 24 for AqpZ−. (D) Cells were grown in LB (0.44 Osmol/kg) for 15 hours and transferred into LB supplemented with 620 mM sucrose. Averaged traces were obtained from 12 cells for AqpZ+ and 15 cells for AqpZ−.

In order to obtain the time rate of *E.coli* cytoplasmic water efflux under more standard conditions, we monitored a cytoplasmic-expressed fluorescent protein in single cells subjected to hyperosmotic shock in rich medium containing a number of osmoprotectants. For each experiment, cells were transferred from LB media to LB supplemented with different amounts of sucrose in a flow chamber. Each cell was observed for 20 min after shock with a sampling rate of one frame per second to obtain a data set comprising 854 individual cells. Figure 2A shows normalized averaged volume traces for each sucrose shock (there are approximately 100 cells for each shock condition). We observe two phases to the reduction in volume: an initial fast decrease and a later slower stage. The rate of volume reduction in the faster phase, *dV_n_/dt*, can be as high as 8% per second and depends on the magnitude of the osmotic shock (Figure 2B). The remaining volume reduction, most clearly visible for the two highest shocks, proceeds on a much slower time scale. We attribute this slower phase to rehydration of the periplasm upon sucrose penetration into the periplasmic space as observed in Figure 1.

### Aquaporin Z is not required for fast water efflux

The molecular mechanisms underlying the fast movement of water across the *E.coli* cell membrane has been difficult to pin down. In particular, while several reports have indicated that aquaporin Z (AqpZ) is involved in water transport, studies looking at when AqpZ is expressed and whether it increases membrane water permeability have been inconclusive. Electron microscopy studies reported that the deletion of *aqpZ* increased water efflux times from ∼10 seconds to ∼100 seconds [28]. These experiments were performed on cells grown to late exponential phase based on a study that showed increased expression of plasmid-encoded *aqpZ* during this phase [29]. This same study also suggested that the expression of *aqpZ* is further increased during growth in low-osmolarity media. However, other studies have found that the expression of *aqpZ* and its effect on water permeability is largely present only in late stationary phase [30], [31].

To determine the role of AqpZ during water efflux, we measured the cytoplasmic cell volume reduction in strains containing and lacking the AqpZ protein under various conditions (see *Materials and Methods*). Even in the absence of the aquaporin, we observed rapid water efflux at a rate of 7–11% per second to approximately the same final volume (Fig. 2C,D,E). Specifically, upon the addition of 600 mM sucrose, the timescale for water efflux was equally fast in the AqpZ+ and AqpZ− strains under various growth and osmotic conditions in contrast to previously published estimates. Interestingly, the magnitude of volume reduction appeared to depend on growth phase and medium osmolarity. In stationary phase, we observe a prominent second, longer timescale of volume reduction present in both the AqpZ+ and AqpZ− strains. Cells shrink more in the absence of AqpZ in this growth condition. This was the only observed difference between the two strains. From these data, we conclude that AqpZ is not required for fast water efflux upon hyperosmotic shock. Further experiments will be needed to systematically study the role of AqpZ in water efflux during hyperosmotic shock.

### Cell volume recovery is complex and occurs on multiple time scales

**Figure 3:**
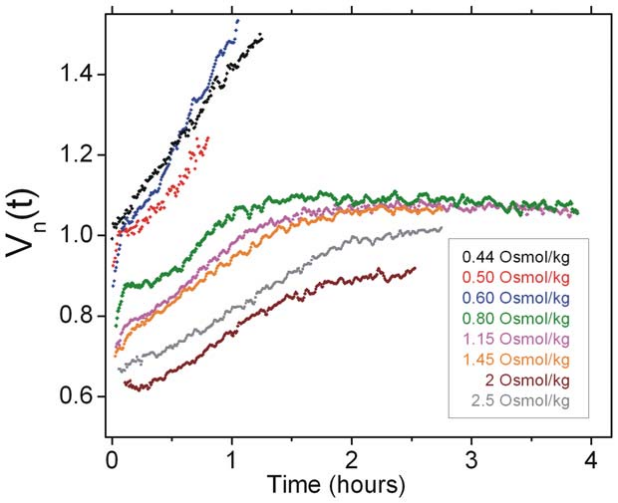
Volume recovery is complex and required for growth. Averaged recovery traces shown for different shock magnitudes (V_n_(t)). The initial 10 minutes of data is recorded at 1 Hz frame rate, subsequent data is recorded at a frame every 30 seconds. For each cell in a given data set, the recovery trace was normalized by the initial volume, aligned at the time of hyperosmotic shock and resampled every 30 seconds. The average trace was computed from these normalized and aligned data sets. The following conditions are shown: 0.44 Osmol/kg data (N = 9), 0.5 Osmol/kg (N = 4), 0.6 Osmol/kg (N = 22), 0.8 Osmol/kg (N = 6), 1.15 Osmol/kg (N = 28), 1.45 Osmol/kg (N = 16), 2 Osmol/kg (N = 12) and 2.5 Osmol/kg (N = 5).

Previous studies of osmolyte uptake have largely focused on cell growth in hyperosmotic media [11], [32]. In contrast, studying the events immediately upon hyperosmotic shock has been harder to achieve. To look at cell recovery response *in vivo* and in rich media immediately upon shock, we observed cytoplasmic volume over the course of several hours. Figure 3 shows averaged recovery traces for sucrose shocks of different magnitudes between 0.50 and 2.5 Osmol/kg.

**Figure 4:**
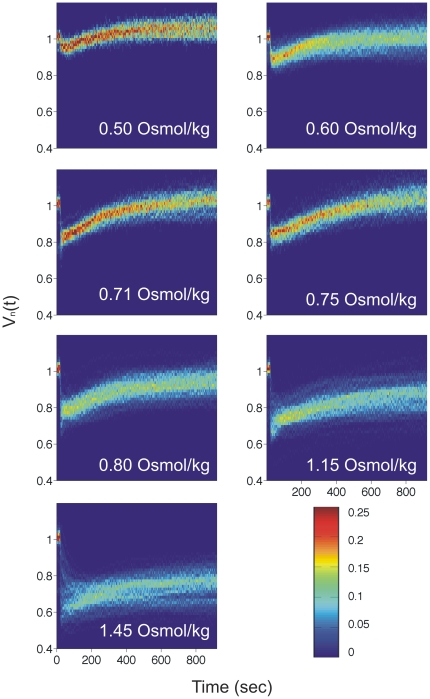
2D Probability density function (PDF) of the recovery traces for a given shock magnitude. Approximately 100 cells per shock magnitude are includes. PDFs are calculated from normalized and aligned data sets as in Figure 3. Cells were shocked by transferring them from LB into LB and a given amount of sucrose. Post shock osmolality is given in each panel.

All of the individual cell traces are given together with the average traces in (Figure S1). Recovery response depends on the magnitude of the shock. For shocks smaller than or equal to 0.60 Osmol/kg, the cells quickly recover their original cell volume, *V_n_* = 1, and proceed to grow. In contrast, for shocks between 0.80 and 1.45 Osmol/kg, this fast phase of recovery is followed by a slower phase that brings the cell volume to near its initial value. The slower phase asymptotes to an indefinite pause that can last several hours during which no further recovery or growth is observed. For the 2 Osmol/kg and 2.5 Osmol/kg shocks, only the slower phase of recovery is observed. In this case, the cell volume never reaches the initial value, even after several hours. Remarkably, cells undergoing the fast phase of recovery exhibited a very small amount of cell-to-cell variability (Figure 4).

For shocks larger then 2.5 Osmol/kg, cells were unable to recover (Figure S2). Transfer of these cells back to normal LB medium results in immediate volume recovery and the resumption of growth. We therefore conclude that these largest shocks produce a free-energy barrier to osmoprotectant accumulation that cannot be overcome by the various pumps in the cell.

### Two-phase recovery involves protein synthesis

**Figure 5:**
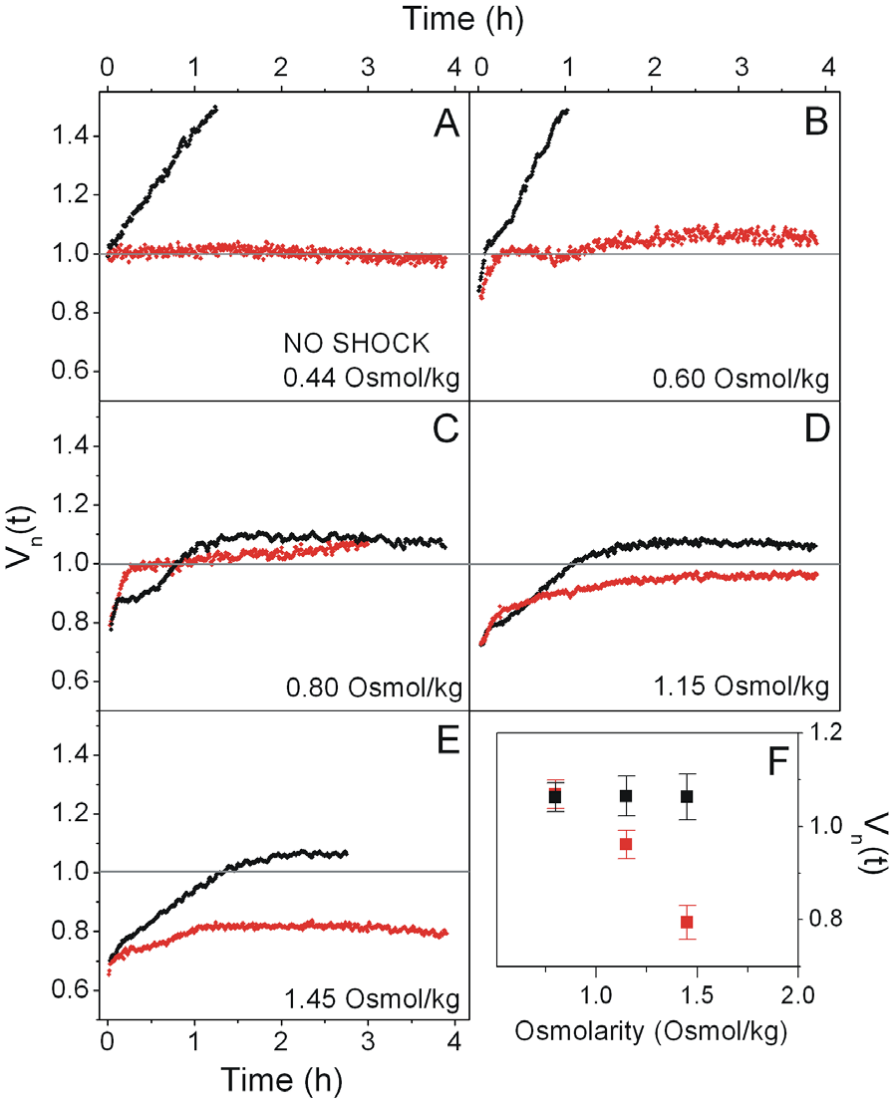
Slow recovery requires protein synthesis. (A)–(E) Averaged recovery traces shown for different shock magnitudes (V_n_(t)). For each shock, the recovery trace in the presence (red) and absence (black) of the drug chloramphenicol is shown. Data was recorded and analyzed as in Figure 3. 0.44 Osmol/kg (no shock, N = 10), 0.6 Osmol/kg (N = 5), 0.8 Osmol/kg (N = 5), 1.15 Osmol/kg (N = 20) and 1.45 Osmol/kg (N = 10). (F) Final volume, calculated as the average of the last 15 minutes of data in (A–E), versus shock magnitude. Error bars are standard error of the mean.

To determine the influence of protein synthesis on the different phases of recovery, we measured the recovery of cells during hyperosmotic shock in the presence of chloramphenicol, a drug that inhibits peptidyl transferase activity of the ribosome [33]. Control experiments in which we quantified cell volume over time without osmoshock but in the presence of chloramphenicol indicate that volume remains constant and growth is inhibited as expected (Figure 5A). For a small sucrose shock, volume recovery is not affected by the presence of the drug whereas subsequent growth is abolished (Figure 5B), indicating that the initial phase of volume recovery does not require protein synthesis and is more likely due to the uptake of osmolytes by protein transporters that are already present in the cell. For higher sucrose shocks, the transition between the two phases of recovery is not observed in the presence of chloramphenicol (Figure 5C). Instead, the cells exhibit a more monotonic recovery that asymptotes to the final volume. Thus, the secondary recovery phase requires protein synthesis. For the highest sucrose shocks, recovery is significantly inhibited by chloramphenicol, indicating that protein-synthesis-driven pathways are essential for this process (Figure 5C–E). In these cases, the final volume is significantly less than what the cell can achieve when protein synthesis is active (Figure 5F).

## Discussion

Looking at the cell's passive response in real time has enabled us to observe previously unseen shape changes. Immediately upon osmotic shock, water efflux causes a reduction of the cell and cytoplasmic radii but little change in the lengths. We find that the rate of volume reduction is ∼10% per second and that during this initial shrinking the inner membrane is not detached from the cell wall. This phase of volume reduction lasts for ∼5 seconds and the *E.coli* Aquaporin Z protein is not required for fast water efflux. Immediately after shrinking, we observe an increase in the cell radius and attribute this to diffusion of sucrose into the periplasm of the cell. As the total cell radius increases, cytoplasmic cell length decreases such that the inner membrane detaches from the cell wall mostly at the cell poles. The final result is a typical plasmolyzed cell as seen in electron microscopy experiments [24], [25], [27], [34]–[37]. As with the fast phase of shrinking, AqpZ played no significant role in the rate of volume reduction on longer time scales. The only difference we found between cells with and without AqpZ was the value of *V_min_* upon hyperosmotic shock for the cells grown to late stationary phase. We find that AqpZ^+^ cells shrink less when compared to the AqpZ^−^ strain. It is currently not clear why this might be and further experiments are needed to fully address the role of AqpZ, if any, in the passive flux of water through the *E. coli* inner membrane.

In rich media, volume adaptation upon hyperosmotic shock shows characteristic phases that depend on the magnitude of the shock. On the fast time scales, lasting ∼20 minutes, recovery is likely due to the import of osmolytes by transporters that are constitutively expressed during normal growth. Larger shocks require an even larger accumulation of solutes into the cytoplasm. This happens during a slower phase of recovery and requires protein synthesis. We hypothesize that the typical level of transporters that are present during normal growth are capable of regulating the turgor pressure during small changes to the external osmolarity, shocks of magnitude less than ∼0.6 Osmol/kg, that may normally be encountered in the environment. Larger perturbations, which are encountered far more infrequently in the wild, require the synthesis and action of secondary recovery pathways that are only used in extreme circumstances.

In addition, protein synthesis-driven mechanism seems to switch the fast recovery phase off while at the same time inducing the slower phase. This is most readily seen in Figure 5C, where the volume plateaus in time after ∼20 minutes without chloramphenicol, but keeps increasing for another ∼15 minutes when protein synthesis is inhibited. Previous research suggests that the increase in cytoplasmic potassium concentration during the fast recovery phase triggers the synthesis of potassium efflux channels. Efflux of potassium is then observed to accompany trehalose synthesis such that the cell exchanges one osmoprotectant for another [32]. It is reasonable to predict, therefore, that the slower phase of volume recovery we observe corresponds to the export of potassium and the accumulation of trehalose.

A number of differences exist between the experiments referenced above and our experimental protocol, which must also be considered. First, trehalose accumulation is observed on the order of 30 min after shock, while the slow recovery we observe lasts several hours [32]. Second, the growth conditions and shocking media, including media osmolarity and potassium concentrations, varied between the different protocols. Third, a Kdp- strain was used in that work, which at the osmolality and potassium concentrations they probed was indistinguishable from the wild type. In LB media, this is not the case and we expect protein-synthesis to be responsible for the production of nascent transporters, such as the Kdp potassium pump [38], [39]. Furthermore, the secondary transporter ProP is both constitutively expressed and expressed under conditions of osmotic stress as a result of increased internal potassium concentrations [40]. Induction of the *proU* gene is fast and has been observed within ∼4 min after osmotic shock [40]. Future experiments using sucrose and LB media that monitor the cellular concentrations of potassium and trehalose at the single cell level will be needed to further connect these aspects of the recovery and address possible redundancies among the different recovery pathways.

For shocks in the range of 0.50 to 1.45 Osmol/kg the slower recovery phase enables the cells to reach their initial volume, but it is followed by a long pause. Unshocked cells and cells shocked with a small amount of sucrose continue to grow during this same length of time in our experimental chambers indicating that the pause at the end of the slow rate recovery is unlikely due to nutrient limitation. In liquid culture at room temperature and with no shaking, these cells will eventually grow to high density, usually within a day. Therefore at present we do not understand the nature of this pause. It is possible that growth resumes at a later point and that only very few cells continue to grow after larger shocks. Future experiments that will monitor the cell volume changes on even longer time scales can help us understand the observed pause.

For very large sucrose shocks, above 2.5 Osmol/kg, no recovery is observed. These large shocks produce a free-energy barrier to osmoprotectant accumulation during the initial recovery rate. We estimate this maximum energy, ΔG_max_, for potassium pumping using the known external, ∼8 mM, and internal, ∼250 mM, concentrations of potassium for cells grown to exponential phase in LB [41], [42]. A hyperosmotic shock of ∼2.5 Osmol/kg decreases the cytoplasmic volume by about a factor of two, so that the concentration of potassium in non-recovering, shocked cells is estimated to be ∼500 mM. To include the electrostatic energy-contribution for transport of potassium across the inner membrane, we assume the membrane voltage is on the order of 150 mV [43], [44]. Using these values, we calculate a maximum energy of ΔG_max_∼25 kJ mol^−1^, of the same order of magnitude as the maximum energy output of light-powered proteorhodopsin and other cellular pumping systems [45]–[47].

Lastly, we comment that most of the previous research on osmoregulation in *E. coli* used the addition of sodium chloride to produce an external hyperomostic shock [11]–[14], [25], [32]. We chose sucrose because *E.coli* membrane permeability to it is understood. A previous report found drastic differences in potassium uptake depending on the shocking agent [38]. These results are unsurprising. Charged solutes influence the overall energetic state of the cell, including ΔG_max_ for a particular pump in a given condition. Consequently the sequence of events during the initial stage of osmorecovery can be altered. Methods to monitor *E.coli* cells membrane voltage on a single cell level and in real time with high temporal resolution have been recently reported [43], [48]. Simultaneous measurements of cell volume changes and changes in cell energetic state promise to bring additional answers on cells' overall stress response.

High-resolution measurements of cell volume changes upon hyperosmotic shock have given us new insight into the dynamics of cytoplasmic water efflux and cell volume recovery. In a similar manner we hope to extend our measurements to fully understand and answer open questions regarding the sequence of events involved in osmoregulation and other stress responses.

## Materials and Methods

### Bacterial strains

*E.coli* strains YD133 (Δ*FimA*, Δ*FliC*, Δ*FlgE* derived from the K12 strain) with plasmid pWR20 (carrying enhanced green fluorescent protein, EGFP, and kanamycin resistance) and AqpZ− strain with plasmid pWR20 (K12 strain lacking *ApqZ*) were grown from frozen stocks, derived from single colonies in: (1) Luria-Bertani (LB) medium (0.44 Osmol/kg prepared with 0.01 g/ml NaCl) supplemented with 25 µg/ml kanamycin at 37°C with shaking to an optical density (OD) of 0.45–0.65. (2) Low Osmolarity-LB (LB prepared with no sodium chloride, 0.08 Osmol/kg) supplemented with 25 µg/ml kanamycin at 37°C with shaking to an OD of 0.8. (3) LB medium (0.44 Osmol/kg) supplemented with 25 µg/ml kanamycin at 37°C with shaking for 15 hours.

Cells grown to OD of 0.45–0.65 in LB were subsequently kept at room temperature and used for sample preparation for up to 3.5 hours (up to a maximum OD of 0.85), and for up to 30 min in all other conditions.

### Sample preparation

Cells were concentrated 5 times in LB and 0.75 µm diameter latex spheres were added to a final concentration of ∼0.1% wt/vol (Bangs Beads). For total cell volume measurements cells were incubated for 10 min in LB supplemented with 10 µg/ml final concentration FM 4–64 dye prior to concentrating them 5 times. Microscope coverslips were assembled into tunnel slides as described previously [49]. Both cells and microspheres were immobilized on a glass cover slip surface of a tunnel slide as follows: 1% polyethylenimine (PEI) was introduced into the tunnel and incubated for 1 min. Unbound PEI was removed from the tunnel by washing with 100 µl LB twice. Cells with 0.75 µm beads in LB were flown in the tunnel and incubated for 5 min. As with PEI, unbound beads and cells were washed out of the tunnel with LB or the appropriate buffer.

### Microscopy

Cells were observed in epifluorescence and differential-interference contrast (DIC) using a modified Nikon TE2000 microscope as described [50]. At the beginning of each experiment, a field of view with 5 or more flat cells (see Data Analysis) is chosen. In order to maintain the chosen field of view with cells of interest at a fixed *x*, *y* and *z* position during an experiment, we use back-focal-plane (BFP) interferometry [51], [52] on a bead stuck to the coverslip surface. The position of a 0.75-µm sphere (bead) attached to the coverslip surface was detected using an 855-nm diode laser (detector laser, Bluesky Resarch) and a position sensitive detector (PSD, Newfocus). A stuck bead closest to the detector laser position is moved through its focus with a piezo-driven sample stage (Mad City Labs). The center of the detector beam is defined as the midpoint of the single valued range of dimensionless *X* and *Y* signals obtained from the PSD. The bead is then kept fixed in the center of the detector laser in *x*, *y* and *z* using a standard proportional-integral-derivative (PID) feedback algorithm [49] implemented in LabView. Images of cells expressing EGFP were acquired at an exposure time of ∼0.1–0.2 seconds using a 512×512 pixel (16 µm^2^, 80 nm effective pixel size) back-thinned electron-multiplying charge-coupled device camera (Andor Technologies). Frame rate was kept at 1 Hz for recordings of up to 20 min and at a frame every 30 seconds for longer recordings (up to several hours). Cells treated with outer membrane dye FM 4–64 were acquired at an exposure time of ∼0.4–0.8 second with the same camera and a frame rate of 0.6 Hz. During simultaneous outer membrane dye and cytoplasmic EGFP recordings images were acquired one after the other with 0.6 Hz. Trans- and epi-illumination light was shuttered in between image recordings to reduce photobleaching.

### Hyperosmotic shock

To increase the osmolarity of the external environment in the tunnel slide, LB is exchanged for LB supplemented with a defined sucrose concentration in all but one experiment. When investigating the passive response to hyperosmotick shock, cells attached to the coverslip surface of a microscope tunnel slide are first transferred from LB into a Tris-based buffer that is isosmotic to LB. To find the isotonic buffer conditions, cells in LB were transferred into 10 mM Tris-HCl, pH = 7.5, supplemented with different concentrations of sucrose. We then monitored the initial change in cell size upon the addition of this buffer and determined that a buffer containing 150 mM sucrose produced virtually no visible change to the cell size during buffer exchange. To perform a controlled osmotic shock, cells were then transferred from the isotonic buffer to the same buffer supplemented with additional sucrose. Increases in sucrose concentration are expressed as molarities, appropriate osmolalites were calibrated with an osmometer (Osmomat30, Genotec, Germany). For concentrations higher then 700 mM sucrose in LB, crystals no longer formed in the osmometer and we calculated the appropriate osmolalities from known molal concentrations.

### Data analysis

Data analysis was only performed on cells that appeared to be uniformly attached to the cover slip surface, as indicated by a uniform fluorescent intensity of cytoplasmic EGFP across the cell area for a given focal position. In our experience, these flat cells are less likely to move in *x*, *y* or *z* when the media is exchanged, ensuring a more accurate estimate of changes in cell volume. Upon choosing a flat cell, the cell's long axis is manually aligned either vertically or horizontally with the image axis. For the cytoplasmic area measurements a rectangle is chosen around the cell (Image Rectangle). Inside the Image Rectangle, a second rectangle, covering only background pixels near the cell is selected (Background Rectangle). The pixel intensities were extracted from the Image and Background Rectangle and the mean intensity of the Background Rectangle was subtracted from all the pixel values in the Image Rectangle in each individual frame. For each frame, pixel values were normalized and scanned for minimal and maximal value. The pixels whose intensity was above 30% of the difference between the two values was chosen as belonging to the cell cytoplasm. The number of pixels above this threshold was recorded for each frame. Records of cell area versus time, *S_cyto_(t)*, were filtered with a 3-point median filter to remove spurious points. For the total cell area measurements, aligned images were passed through Median Filter of radius 1.5 in ImageJ software (freeware). As before, for each frame pixel values were obtained, mean background intensity subtracted, and frames scanned for minimal and maximal pixel value. The pixels whose intensity was above 40% of the difference between the maximal and minimal pixel value was chosen as belonging to the cell. Outer membrane dye gives two characteristic peaks in the intensity at the edge of the cell, with a small drop in the intensity in the middle. Given that the intensity in the middle of the cell is still large compared to the background despite the drop, the tresholding method gives a contour around the cell for each frame. Pixels inside the contour have been counted to obtain a record in time *S_total_(t)*. Out of focus light from regions of the cell cylinder above and below the focal plane tend to widen the area subtended by the measured contours. We correct for this by numerically calculating the magnitude of the effect using a measured 3D PSF. Cell stuck to the surface was assumed to be a cylinder (2 µm long and 1 µm diameter) capped by two hemispheres (1 µm diameter), and cell area converted to volume 
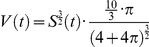
.

Total cell length and radius as well as the length and radius of the cell cytoplasm were calculated by finding the middle pixel column and row for *S_total_(t)* and *S_cyto_(t)*. Three columns on each side of the middle column were used to obtain the average cell length. The same was done for the rows to obtain the average cell radius.

Averaged recovery traces were obtained from traces of each cell in a given data set. For each cell the recovery trace was normalized, such that *V(t) = V(t)/V_0_*, where *V_0_* is an average of the cell volume in the first five seconds of the recording. Normalized recovery traces were aligned at the time of hyperosmotic shock, *t_1_*. For this, *d(V(t))/dt* was calculated using *V(t)* from the beginning of the recording to the time the cell reaches *V_min_*. Then *t_1_ = t_min−1_* where *t_min_* is the time at which *d(V(t))/dt* is minimal and *V_min_* is the average of 10 seconds of data centered on the minimum cell cytoplasmic volume recorded within first 5 min after the hyperosmotic shock. The average trace was then computed from these normalized and aligned data sets. Histograms in Figure 4 were obtained from normalized and aligned data sets. The rate of cytoplasmic volume reduction was calculated as *d(V(t))/dt* from the five seconds immediately following the hyperosmotic shock.

## Supporting Information

Figure S1 **Volume recovery traces of each individual cell for a given shock magnitude.** Averaged recovery traces for different shock magnitudes (V_n_(t)) are given in bold and correspond to the traces in Figure 3. Post shock osmolality and number of cells are given in each panel.
(TIF)

Figure S2 **Normalized volume recovery trace of a cell transferred from LB into LB and 3 molal sucrose and then back into LB.** No recovery was observed for one hour after the initial hyperosmotic shock, at which point a cell was transferred from LB with 3 molal sucrose back into LB. In a different experiment at this shock level, volume was monitored up to 4 hours and no recovery was observed. After returning the cell to LB, an effective hypoosmotic shock, an immediate rehydration of the cell is observed followed by cell growth and division. At this magnitude of the shock, at least 40% of the cells continued growing and dividing upon transition back into LB. 60% of the cells expanded their volume to initial value but failed to grow. Close up of the hyperosmotic (Inset A) and hypoosmotic shock (Inset B). Red arrows in the insets indicate the time of shock. Periods during, and immediately before and after shocks were recorded at 1 Hz frame rate, while the rest of the time a frame every 30 seconds was recorded.
(TIF)

Video S1 **A sequence of raw fluorescent images of a single** ***E.coli*** **cell prior and during a hyperosmotic shock showing fast water efflux.** The cell is transferred from isoosmotic buffer (10 mM Tris-HCl buffer supplemented with 150 mM sucrose) to isoosmotic buffer and 620 mM sucrose. Cytoplasmic volume was marked with the green protein EGFP and total cell volume by the red outer membrane dye FM4-64. Images were acquired at a frame every 1.6 seconds alternating between the cytoplasmic volume and total cell volume.
(MOV)

Video S2 **Composite images constructed from the fluorescent images in Video S1.** Image brightness was adjusted due to photo bleaching and the difference in fluorescence intensity between the red and green channels. Cytoplasmic volume is given in green and total cell volume in red.
(MOV)
