# Supplementary figures and images for: Fast, Multiphase Volume Adaptation to Hyperosmotic Shock by Escherichia coli
Source: PLoS One. 2012 Apr 13;7(4):e35205. doi: 10.1371/journal.pone.0035205 (PMC3325977; doi:10.1371/journal.pone.0035205)

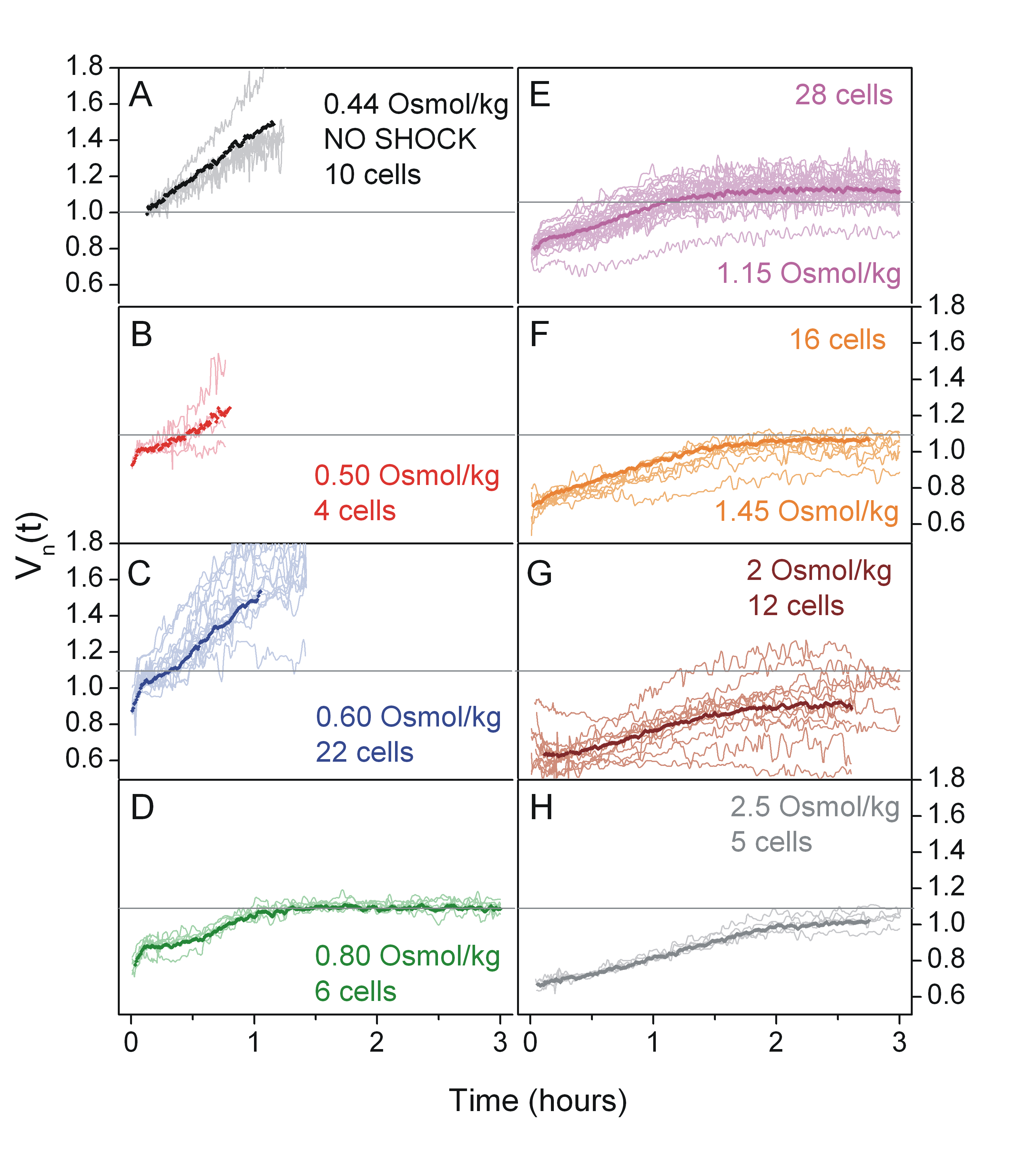

Supplement: Figure S1 — Volume recovery traces of each individual cell for a given shock magnitude. Averaged recovery traces for different shock magnitudes (Vn(t)) are given in bold and correspond to the traces in Figure 3. Post shock osmolality and number of cells are given in each panel. (TIF) [file pone.0035205.s001.tif]

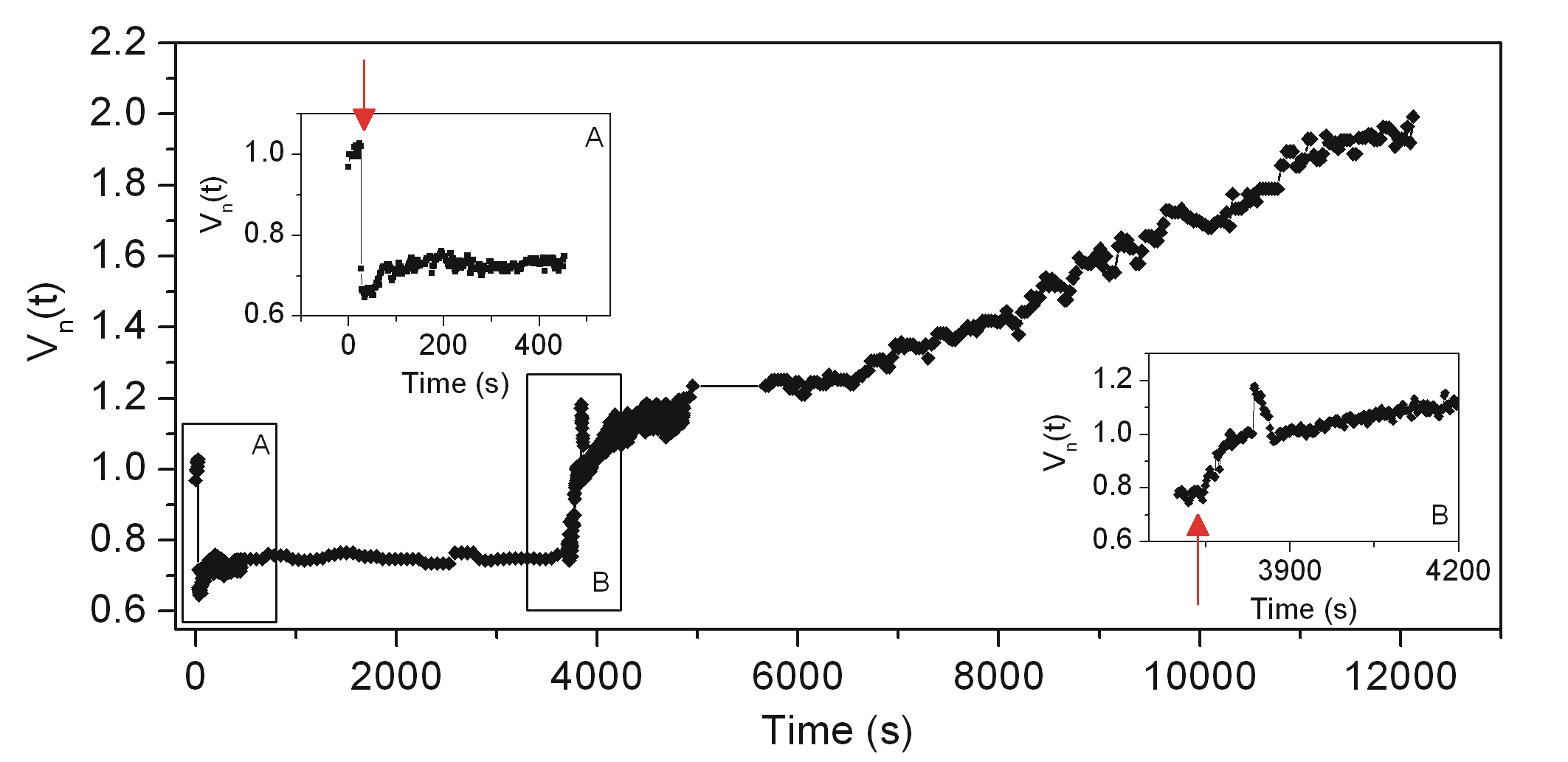

Supplement: Figure S2 — Normalized volume recovery trace of a cell transferred from LB into LB and 3 molal sucrose and then back into LB. No recovery was observed for one hour after the initial hyperosmotic shock, at which point a cell was transferred from LB with 3 molal sucrose back into LB. In a different experiment at this shock level, volume was monitored up to 4 hours and no recovery was observed. After returning the cell to LB, an effective hypoosmotic shock, an immediate rehydration of the cell is observed followed by cell growth and division. At this magnitude of the shock, at least 40% of the cells continued growing and dividing upon transition back into LB. 60% of the cells expanded their volume to initial value but failed to grow. Close up of the hyperosmotic (Inset A) and hypoosmotic shock (Inset B). Red arrows in the insets indicate the time of shock. Periods during, and immediately before and after shocks were recorded at 1 Hz frame rate, while the rest of the time a frame every 30 seconds was recorded. (TIF) [file pone.0035205.s002.tif]
